# Investigating group-velocity-tunable propagation-invariant optical wave-packets

**DOI:** 10.1038/s41598-022-20601-0

**Published:** 2022-09-27

**Authors:** Zhaoyang Li, Yanqi Liu, Yuxin Leng, Ruxin Li

**Affiliations:** 1 Zhangjiang Laboratory, 100 Haike Road, Pudong, 201210 Shanghai China; 2 Institute of Laser Engineering, Osaka University, 2-6 Yamada-oka, Suita, Osaka 565-0871 Japan; 3 Shanghai Institute of Optics and Fine Mechanics, Chinese Academy of Sciences, 390 Qinghe Road, Jiading, 201800 Shanghai China; 4 ShanghaiTech University, 393 Middle Huaxia Road, Pudong, 201210 Shanghai China

**Keywords:** Optics and photonics, Optical physics, Slow light

## Abstract

The group-velocity of the propagation-invariant optical wave-packet generated by the conical superposition can be controlled by introducing well-designed arbitrarily-axisymmetric pulse-front deformation, which permits realizing superluminal, subluminal, accelerating, decelerating, and even nearly-programmable group-velocities. To better understand the tunability of the group-velocity, the generation methods of this propagation-invariant optical wave-packet and the mechanisms of the tunable group-velocity in both the physical and Fourier spaces are investigated. We also have studied the relationship with the recently-reported space–time wave-packet, and this group-velocity-tunable propagation-invariant optical wave-packet should be a subset of the space–time wave-packet.

## Introduction

Controlling the group-velocity of an optical pulse, especially a propagation-invariant optical wave-packet, is an interesting and important research, which has many applications from optics to physics and engineering^1–6^. The Bessel beam is a very famous family of propagation-invariant beams^7,8^, which nearly does not spread out in space during very-long-distance propagation much exceeding the Rayleigh length^9,10^. Apart from the propagation-invariance, the Bessel beam, compared with the Gaussian beam, has many other unique characteristics like self-healing, superluminal (both phase and group velocities), etc.^11–13^, which further enhance its applications^14^. A monochromatic Bessel beam is propagation-invariant in space, while a broadband Bessel wave-packet can maintain its invariant intensity profile in both space and time, which accordingly becomes a very important family of propagation-invariant wave-packets^15–17^. The previous researches show the Bessel wave-packet can be expressed as a coherent superposition of monochromatic Bessel beams of a range of frequencies. The first broadband propagation-invariant wave-packet (X-wave) studied in acoustics by J. Lu et al. can remain its spatiotemporal profile during propagation^15,16^, and one optical version named as the Bessel-X beam has been demonstrated in experiments by P. Saari et al.^17^. Some other forms of the Bessel optical wave-packet such as focus wave modes^18–21^, light-needles^22^, or light-bullets^23^, etc. have been produced by modulating angular/spatial dispersion (frequency-dependent conical angles), polarization, or temporal dispersion, etc. during the coherent superposition of monochromatic Bessel beams with different frequencies. These Bessel optical wave-packets further enhance the unique characteristics, such as wide-ranging-tunable group-velocities, enhanced spatiotemporal propagating-invariance, etc., and bring new opportunities for applications.

Recently, kinds of spatiotemporal coupling methods are widely used to control the propagation or structure of Bessel wave-packets^24–28^, space–time wave-packets^29–38^, flying focuses^39–42^, spatiotemporal optical vortices^43–45^, etc. In our previous works, we have proposed a method to control the group-velocity and group-acceleration of the propagation-invariant optical wave-packet generated by the conical superposition by separating the pulse-front from the phase-front and shaping the pulse-front from a plane into an arbitrarily-axisymmetric distribution, and in simulation superluminal or luminal or subluminal group-velocities, accelerating or uniform-motion or decelerating group-accelerations, and even nearly-programmable group-velocities in a single propagating path have been introduced^46,47^. In Refs.^46,47^, we gave a short explanation of this phenomenon in the physical space. In this paper, referring to the previous works^24–38^, we studied different forms of this group-velocity-tunable propagation-invariant optical wave-packet in both the physical and Fourier spaces, derived the group-velocity equations in two spaces, and systematically explained the mechanism why the group-velocity could be controlled, which would help well understand this wave-packet and explore possible applications. We also discussed the connection with the recently-reported space–time wave-packet by A. Abouraddy et al.^30–37^, which has freely tunable group-velocity and group-acceleration. The space–time wave-packet was firstly localized in the 2-dimensional (2-D) space–time^30–36^, and very recently which has been localized in the 3-D space–time^37^. In essence, this group-velocity-tunable propagation-invariant optical wave-packet should be a subset of the space–time wave-packet. Because, in the Fourier space, the spatiotemporal spectrum of the former lies in that of the latter (or the spatiotemporal spectrum of the former is a part of that of the latter), and in the physical space, the former consequently has some of the spatiotemporal characteristics of the latter, for example tunable group-velocity and group-acceleration.

## Results

### Generation methods

The Bessel beam can be produced by the conical superposition of plane waves, for example by using a thin axicon^48,49^, and the produced Bessel beam has constant superluminal phase- and group-velocities *v*_p_ and *v*_g_ in the vacuum^13^

1 $$\frac{{v}_{p}}{c}=\frac{{v}_{g}}{c}=\frac{1}{cos\alpha },$$

where, *c* is the light speed in the vacuum and *α* is the half conical-angle for the conical superposition, i.e., the thin axicon induced propagating direction changes of plane waves with respect to the optical axis (*z*-axis).

**Figure 1:**
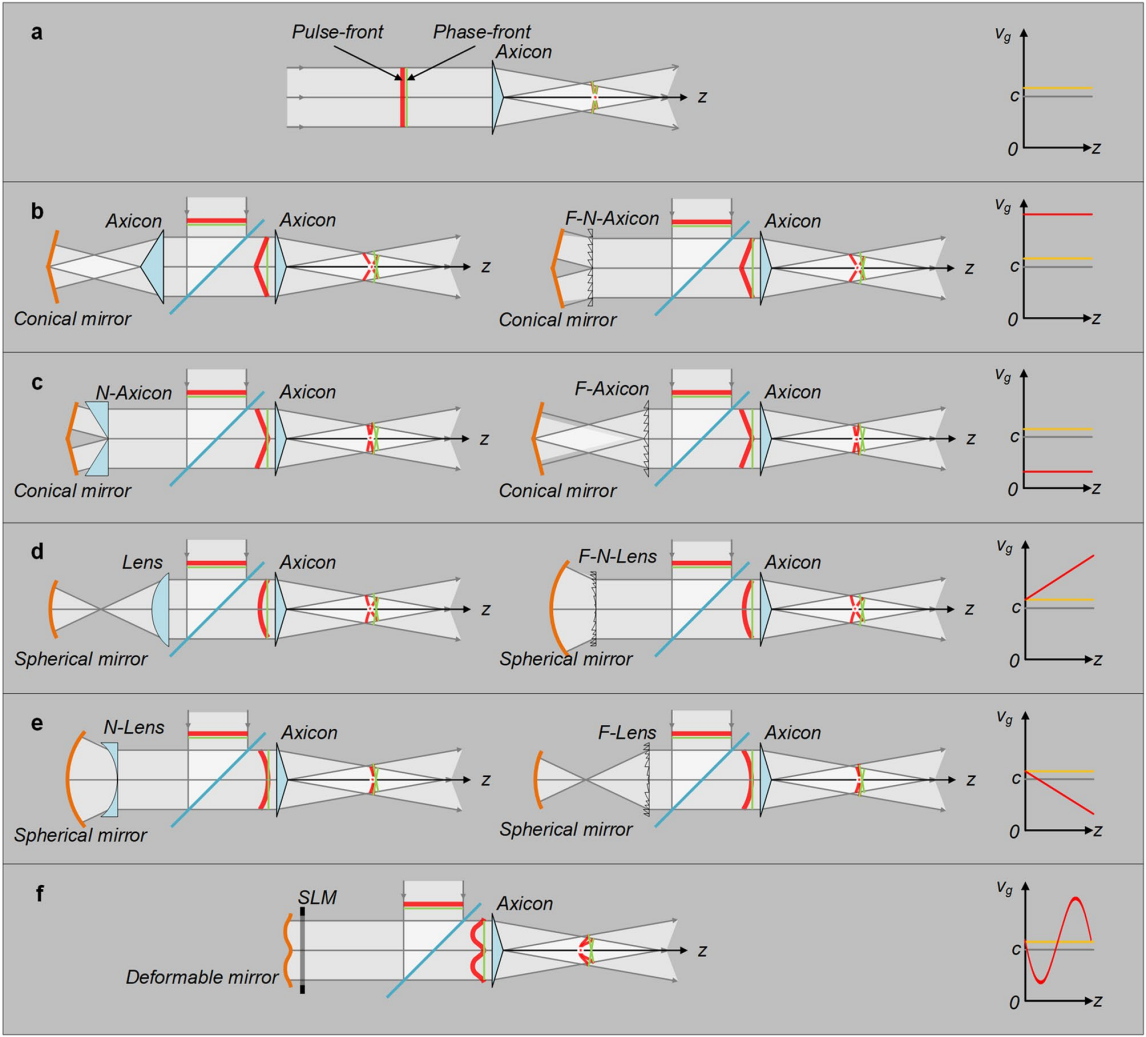
Axicon generated propagation-invariant optical wave-packets for different pulse-front deformations. (**a**) Without any pulse-front deformation, propagation-invariant optical wave-packet has a constant slightly-superluminal group-velocity (yellow curve), and light speed *c* in vacuum is given for reference (gray curve); (**b**) setups and generated concave-conical pulse-front deformation, propagation-invariant optical wave-packet has a superluminal group-velocity (red curve); (**c**) setups and generated convex-conical pulse-front deformation, propagation-invariant optical wave-packet has a subluminal group-velocity (red curve); (**d**) setups and generated concave-spherical pulse-front deformation, propagation-invariant optical wave-packet has an accelerating group-velocity (red curve); (**e**) setups and generated convex-spherical pulse-front deformation, propagation-invariant optical wave-packet has a decelerating group-velocity (red curve); and (**f**) setup and generated axisymmetric complex pulse-front deformation, propagation-invariant optical wave-packet has a variable group-velocity. *F-Axicon* Fresnel axicon, *F-N-Axicon* Fresnel negative axicon, *N-Axicon* negative axicon, *F-Lens* Fresnel lens, *F-N-Lens* Fresnel negative lens, *N-Lens* negative lens, *SLM* spatial light modulator. Figure generated by Microsoft® Visio® 2013^50^.

Figure 1a shows when all frequencies of the input pulsed beam are plane waves and overlap with one another perfectly, the pulse-front (pulse-peak across the beam aperture) overlaps with the phase-front (wave-front of the center angular frequency) perfectly, and the produced propagation-invariant optical wave-packet by an ideal thin axicon has a constant slightly-superluminal group-velocity as illustrated by the yellow line in the *v*_*g*_-*z* plot that can be well described by Eq. (1). However, when we deform the pulse-front of the input pulsed beam from a plane into an arbitrarily-axisymmetric distribution while keeping the plane phase-front unchanged, the propagation of the produced propagation-invariant optical wave-packet would be changed.

**Figure 2:**
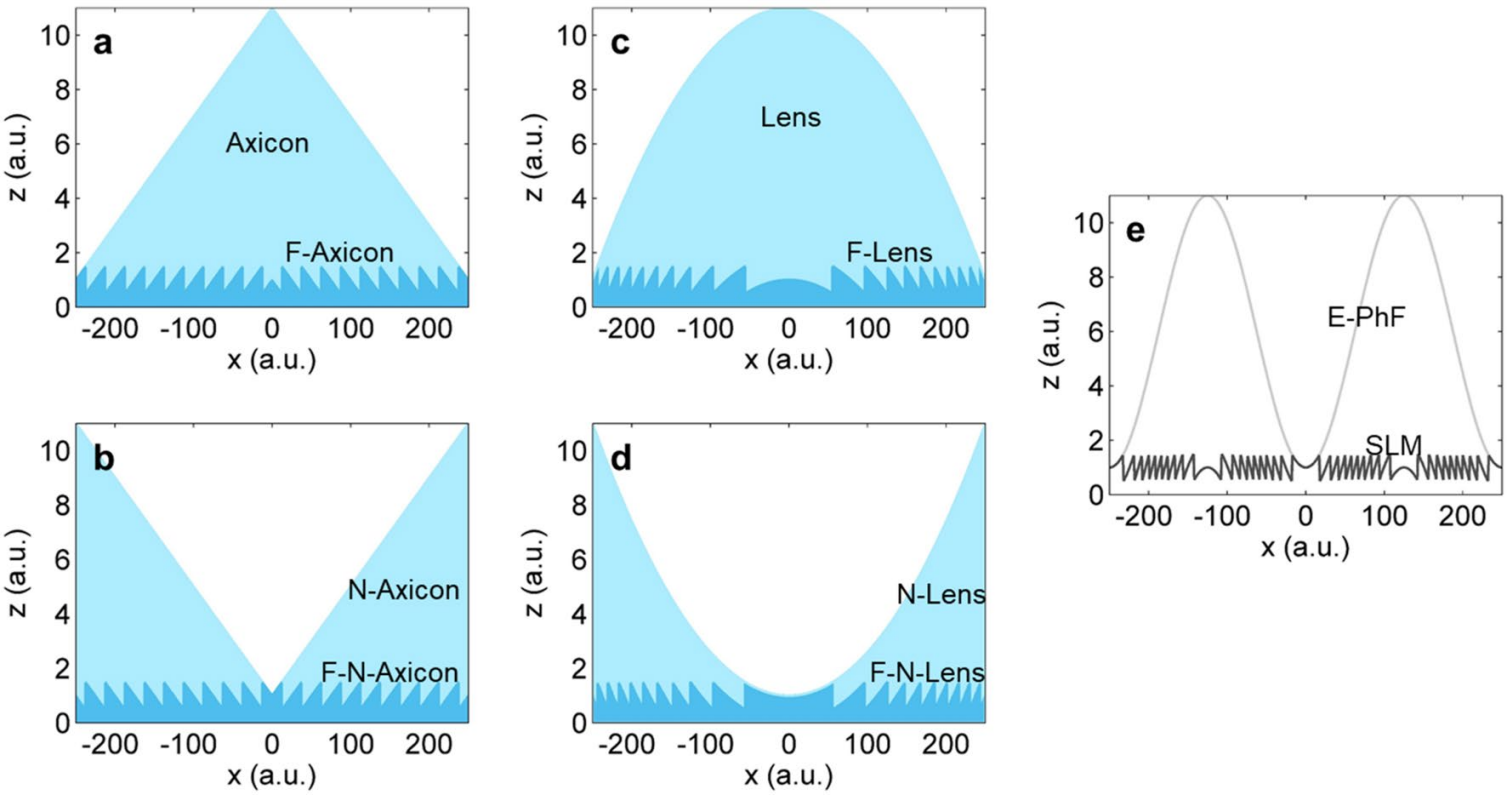
Fresnel optics and SLM. (**a**) Axicon and Fresnel axicon (F-Axicon); (**b**) negative axicon (N-Axicon) and Fresnel negative axicon (F-N-Axicon); (**c**) lens and Fresnel lens (F-Lens); (**d**) negative lens (N-Lens) and Fresnel negative lens (F-N-Lens); and (**e**) SLM’s phase modulation and its equivalent phase-front (E-PhF). SLM, spatial light modulator. Figure generated by Microsoft® Visio® 2013^50^.

We firstly discuss the axisymmetric pulse-front tilt. Figure 1b shows when the deformed pulse-front is concave-conical, the group-velocity of the produced propagation-invariant optical wave-packet increases to a constant superluminal as illustrated by the red line in the *v*_*g*_-*z* plot. The combination of a conical mirror and an axicon or a Fresnel negative axicon (see Fig. 2b) can produce such a concave-conical pulse-front, where the conical mirror retroreflects the pulsed beam and accordingly keeps the plane phase-front unchanged and the axicon or the Fresnel negative axicon introduces bigger and smaller group delays at the beam center and edges, respectively. Figure 1c shows when the deformed pulse-front is convex-conical, the group-velocity of the produced propagation-invariant optical wave-packet decreases to a constant subluminal as illustrated by the red line in the *v*_*g*_-*z* plot. The combination of a conical mirror and a negative axicon or a Fresnel axicon (see Fig. 2a) can produce such a convex-conical pulse-front, where the conical mirror still retroreflects the pulsed beam and keeps the plane phase-front unchanged and the negative axicon or the Fresnel axicon introduces smaller and bigger group delays at the beam center and edges, respectively.

We secondly discuss the axisymmetric pulse-front curvature. Figure 1d shows when the deformed pulse-front is concave-spherical, the group-velocity of the produced propagation-invariant optical wave-packet accelerates linearly from slightly-superluminal [governed by Eq. (1)] to increased-superluminal during propagation as illustrated by the red line in the *v*_*g*_-*z* plot. The combination of a spherical mirror and a lens or a Fresnel negative lens [see Fig. 2d] can produce such a concave-spherical pulse-front, where the spherical mirror retroreflects the pulsed beam and keeps the plane phase-front unchanged and the lens or the Fresnel negative lens introduces bigger and smaller group delays at the beam center and edges, respectively. Figure 1e shows when the deformed pulse-front is convex-spherical, the group-velocity of the produced propagation-invariant optical wave-packet decelerates linearly from slightly-superluminal [governed by Eq. (1)] to reduced-subluminal during propagation as illustrated by the red line in the *v*_*g*_-*z* plot. The combination of a spherical mirror and a negative lens or a Fresnel lens (see Fig. 2c) can produce such a convex-spherical pulse-front, where the spherical mirror retroreflects the pulsed beam and keeps the plane phase-front unchanged and the negative lens or the Fresnel lens introduces smaller and bigger group delays at the beam center and edges, respectively.

We lastly discuss the axisymmetric complex pulse-front deformation. Figure 1f shows when the deformed pulse-front has an axisymmetric complex profile, e.g., axisymmetric cosine-function-like profile, the group-velocity of the produced propagation-invariant optical wave-packet has a sine-function-like variation between reduced-subluminal and increased-superluminal during propagation as illustrated by the red curve in the *v*_*g*_-*z* plot. The combination of a deformable mirror and a spatial light modulator (SLM) (see Fig. 2e) can produce an arbitrarily complex pulse-front, where the deformable mirror shapes both the phase-front and the pulse-front, i.e., no deviation between them, and the SLM corrects the shaped phase-front back to a plane while keeps the shaped pulse-front unchanged^51^.

In summary, when above pulse-front-deformed (phase-front-unchanged) pulsed beams are injected into an ideal thin axicon for generating propagation-invariant optical wave-packets, the result is: the propagating direction of the produced wave-packet is along the *z*-axis and determined by the unchanged plane phase-front, while the propagation forms, including the group-velocity and the group-acceleration, are changed and dominated by the deformed axisymmetric pulse-front, which theoretically can be arbitrarily controlled.

In the above method, the separation of the pulse-front from the phase-front is based on the difference between the group-velocity and the phase-velocity in optics. In transmission optics, e.g., those given in Fig. 2a–d, the pulse-front is delayed in time with respect to the phase-front due to a slow group-velocity and a fast phase-velocity in the normal dispersion medium^52^. Because the thickness of the transmission medium varies across the beam aperture, after collimation by a matched retroreflection mirror, although the phase-front is changed back to a plane, the pulse-front has a deformed spatiotemporal profile which is determined by the shape of the transmission optics (see the first column in Fig. 1b–e). Similarly, if using the anomalous dispersion medium, the pulse-fronts in the first column of Fig. 1b–e would have opposite spatiotemporal deformations. In Fresnel optics, e.g., those given in Fig. 2a–d, the diffraction structure can shape the phase-front like their corresponding transmission optics but almost have no influence on the pulse-front^52^, resulting in the separation between the pulse-front and the phase-front. Similarly, after collimation, the phase-front is changed back to a plane, while the pulse-front is deformed which has an opposite spatiotemporal profile as the corresponding transmission optics (see Fig. 1b–e). Figure 2e shows the SLM is an arbitrarily controllable Fresnel element and can generate an arbitrarily complex phase-front while almost keeping the pulse-front unchanged, and then the combination of the SLM and a matched deformable mirror can deform the pulse-front into an arbitrary spatiotemporal profile and keep the phase-front flat.

### Mechanism in the physical space

In the physical space, a monochromatic Bessel beam in the cylindrical coordinates *ρ*-*φ*-*z* generated by the conical superposition of a monochromatic plane wave is given by^19^

2 $$E_{B} \left( {\rho ,z,k,\alpha } \right) = J_{0} \left( {k\rho sin\alpha } \right)exp\left[ {i\left( {kzcos\alpha - \omega t} \right)} \right],$$

where, *ρ*, *φ*, and *z* are the transverse length, angle, and longitudinal length coordinates, respectively, *t* is the time, *k* is the wavenumber (*k* = *ω*/c in the vacuum), *ω* is the angular frequency, *J*_0_ is the zeroth-order Bessel function of the first kind, and *α* is the half conical-angle (i.e., propagating directions of plane waves with respect to the *z*-axis for conical superposition). Equation (2) shows both the phase- and group-velocities are given by Eq. (1). Because the half conical-angle *α* usually is only slightly larger than 0 (typically 0 < *α* < 10°) for producing a long-distance propagating Bessel beam, the phase- and group-velocities are slightly-superluminal (typically *c* < *ν*_p_ = *ν*_g_ < 1.015*c*).

**Figure 3:**
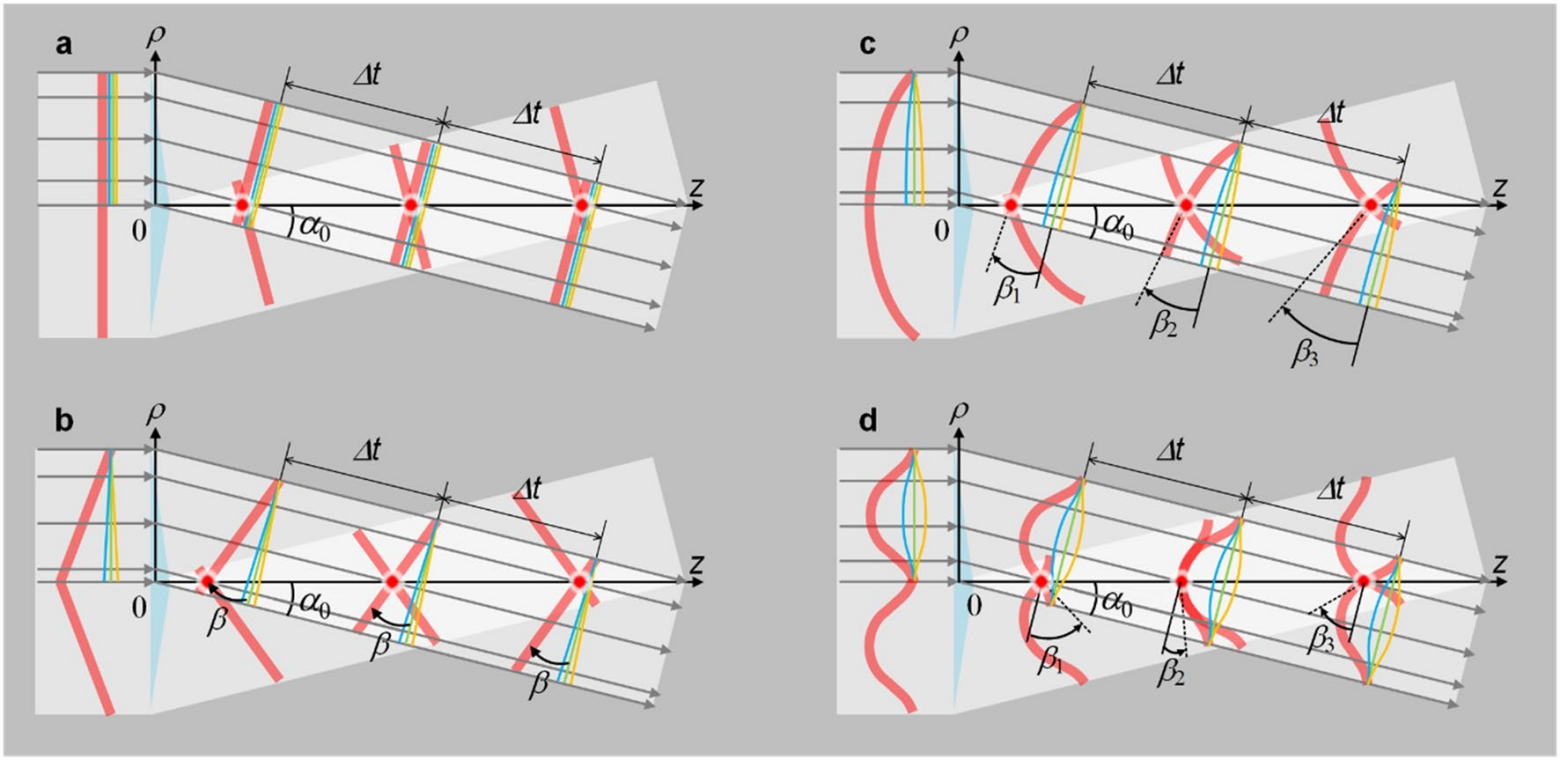
Influence of pulse-front deformation of input pulsed beam on instantaneous group-velocity of produced propagation-invariant optical wave-packet. Because distribution is axisymmetric about *z*-axis, only upper input half beam for conical superposition is illustrated. Red thick curve denotes pulse-front; blue, green and yellow curves denote wave-fronts of the highest, center and lowest frequencies, and that of center frequency is defined as phase-front; *α*_0_ is half conical-angle for phase-front; *β* is pulse-front tilt angle with respect to phase-front; and ∆*t* is a fixed time gap during propagation. (**a**) Without pulse-front deformation, instantaneous group-velocity is only determined by *α*_0_. With pulse-front deformation, instantaneous group-velocity is determined by both *α*_0_ and *β*, and *β* is constant for (**b**) a tilted pulse-front, linearly increases for (**c**) a spherical pulse-front, and periodically varies for (**d**) a cosine-function-like pulse-front. Figure generated by Microsoft® Visio® 2013^50^.

Replacing the monochromatic wave by a plane pulsed beam, the Bessel beam becomes a Bessel wave-packet, which can be presented as the coherent superposition of a series of monochromatic Bessel beams with different frequencies *ω*^26^

3 $${E}_{BWP}\left(\rho ,z,t,{\alpha =\alpha }_{0}\right)=\int dkS\left(k\right){E}_{B}\left(\rho ,z,k,{\alpha =\alpha }_{0}\right),$$

where, *S*(*k*) is the spectral amplitude and *α*_0_ is a frequency-independent constant half conical-angle. Because *α*_0_ is frequency-independent, the phase- and group-velocities are still same and also given by Eq. (1). In geometrical optics, Fig. 3a shows when an ideal thin axicon is located at the *ρ*-plane, the propagation of only the upper input half beam in the lateral plane is illustrated due to the axisymmetric distribution about the *z*-axis. The produced propagation-invariant optical wave-packet, i.e., the intersection of the pulse-fronts (group-velocity), moves along the *z*-axis and perfectly overlaps with the intersection of the phase-fronts (phase-velocity), that is the group- and phase-velocities have no difference and are well governed by Eq. (1).

When the half conical-angle becomes frequency-dependent *α*_*ω*_ (or wavenumber-dependent *α*_*k*_), angular dispersion appears, and the Bessel wave-packet is then given by^19^

4 $${E}_{BWP}\left(\rho ,z,t\right)=\int dk\underset{0}{\overset{\pi }{\int }}d\alpha A\left(k,\alpha \right){E}_{B}\left(\rho ,z,k,\alpha \right),$$

where, *A*(*k*, *α*) denotes the spatio-spectral correlation and can be presented by the Dirac function as^19^

5 $$A\left(k,\alpha \right)=S\left(k\right)\delta \left(\alpha -{\alpha }_{\omega }\right).$$

The frequency-dependent half conical-angle (angular dispersion) would separate the pulse-front from the phase-front resulting in a pulse-front tilt angle^21,53,54^. We define *dα*/*dω* as the conical-angle dispersion and *β* as the pulse-front tilt angle with respect to the phase-front, and both are axisymmetric about the *z*-axis and satisfy the relationship

6 $$tan\beta = \omega_{0} \frac{d\alpha }{{d\omega }},$$

where *ω*_0_ is the center angular frequency. Figure 3b illustrates a typical case of an increased-superluminal group-velocity: within the same propagating time periods as shown in Fig. 3a, the intersection of the pulse-fronts has a longer propagating length than the intersection of the phase-fronts, the latter (phase-velocity) can still be well described by Eq. (1), while the former (group-velocity) is governed by the revised group-velocity equation^46^

7 $$\frac{{v}_{g}}{c}=\frac{cos\beta }{cos\left({\alpha }_{0}+\beta \right)},$$

where both the half conical-angle *α*_0_ (determined by the phase-front) and the pulse-front tilt angle *β* (dominated by the conical-angle dispersion *dα*/*dω*) can influence the group-velocity *v*_g_, i.e., the pulse-front tilt angle *β* becomes another degree of freedom to control the group-velocity. This group-velocity changed wave-packet is also known as the MacKinnon wave-packet^26^ or the baseband space–time wave packet^32^.

In this paper, we define the clockwise rotation from the wave-front of a lower frequency to that of a higher frequency (for the input upper half beam) as the positive conical-angle dispersion *dα*/*dω* and the clockwise rotation from the phase-front to the pulse-front (for the input upper half beam) as the positive pulse-front tilt angle *β*.

Next, we consider a general form, when the pulse-front tilt angle is space-dependent across the beam aperture (but still axisymmetric about the *z*-axis), the group-velocity varies during propagation and can be described by

8 $$\frac{{v}_{g}\left(z\right)}{c}=\frac{cos\beta \left(\rho \right)}{cos\left[{\alpha }_{0}+\beta \left(\rho \right)\right]},$$

where, *ν*_g_(*z*) is a variable group-velocity along the *z*-axis and *β*(*ρ*) is a variable pulse-front tilt angle along the *ρ*-axis at the input. *β*(*ρ*) can also be written as a variable pulse-front tilt angle *β*(*z*) at the *z*-axis, and the transverse and the longitudinal coordinates satisfy the conical superposition relationship *ρ*/*z* = tan*α*_*0*_. Figure 3c illustrates when the input has a concave-spherical pulse-front deformation, from the beam center to the beam edge the pulse-front tilt angle *β*(*ρ*) increases from zero to the maximum, and the instantaneous group-velocity *ν*_g_(*z*) of the propagation-invariant optical wave-packet (the intersection of the pulse-fronts) is increasing, showing accelerating group-velocity. Figure 3d illustrates a more complex case that the input has an axisymmetric cosine-function-like pulse-front deformation, accordingly the pulse-front tilt angle *β*(*ρ*) varies periodically from the beam center to the beam edge, and the instantaneous group-velocity *ν*_g_(*z*) of the propagation-invariant optical wave-packet (the intersection of the pulse-fronts) also varies periodically during propagation along the *z*-axis, showing periodically variable (decelerating-accelerating-decelerating here) group-velocity. All of these phenomena can be approximately described by Eq. (8).

It is worth noting that here the geometrical optics approximation is considered and both diffraction and dispersion-induced distortion are neglected. When the pulse-front tilt angle *β*(*ρ*), accordingly the conical-angle dispersion *dα*(*ρ*)/*dω*, is too large, these two factors would become too serious to destroy the propagation-invariance and break the geometrical approximation of the propagation-invariant optical wave-packet.

### Mechanism in the Fourier space

P. Saari et al. have studied localized (propagation-invariant) waves in the Fourier space *k*_*⊥*_-*k*_*z*_-*k*^26–28^, where, *k*_*⊥*_ and *k*_*z*_ denotes the wavevector component along the transverse and the longitudinal coordinate, respectively, and *⊥*-axis can be *x*- or *y*-axis in the Cartesian coordinates. The spatiotemporal spectrum of a propagation-invariant wave-packet must be the intersecting curve on the light-cone by a spectral plane parallel to the *k*_*⊥*_-axis and its projection onto the *k*_*z*_-*k* plane is a straight line with a slope

9 $$tan\theta =\frac{{v}_{g}}{c},$$

where *θ* is the tilt angle of the spectral plane.

The spatiotemporal spectrum of a propagation-invariant wave-packet is solved by the simultaneous equations^32^

10 $${k}_{\perp }^{2}+{k}_{z}^{2}={k}^{2},$$

and

11 $$\left(k-{k}_{v}\right)=\left({k}_{z}-{k}_{v}\right)tan\theta ,$$

where, Eq. (10) is the light-cone in the vacuum, Eq. (11) is a spectral plane parallel to the *k*_*⊥*_-axis, *k*_*⊥*_ = *k*⋅sin*α* and *k*_*z*_ = *k*⋅cos*α* are the transverse and longitudinal components of *k*, *α* is the direction of *k* in the *k*_*⊥*_-*k*_*z*_ plane (i.e., half conical-angle in the physical space), and *k*_*v*_ is the wavenumber of the vertex of the spatiotemporal spectrum. Refer to Ref.^32^, in Eq. (11) the vertex of the spatiotemporal spectrum is defined as the intersecting point between the spatiotemporal spectrum and the plane *k*_*z*_-*k* (or *k*_*⊥*_ = 0) at the positive direction of the *k*_*z*_-axis, and its coordinates are (*k*_*⊥*_, *k*_*z*_, *k*) = (0, *k*_*v*_, *k*_*v*_), where *k*_*v*_ > 0. Because the spectral plane is always parallel to the *k*_*⊥*_-axis, its tilt angle *θ* is defined with respect to the positive direction of the *k*_*z*_-axis.

A. Abouraddy et al. have used this Fourier space method to investigate their space–time wave-packet^30–37^, and by positioning a SLM at the Fourier plane of a grating 4-f setup (pulse shaper), the spatiotemporal spectrum (including tilt angle and shape) on the light-cone intersected by the spectral plane, and accordingly the propagation characteristic, has been arbitrarily controlled.

Here, we also use this Fourier space method to study the group-velocity-tunable propagation-invariant optical wave-packet generated by the conical superposition for comparison with the result obtained in the physical space. By solving the simultaneous Eqs. (10) and (11), the spatiotemporal spectrum is given by

12 $$cos\alpha =\frac{{\omega }_{v}+\left(\omega -{\omega }_{v}\right)cot\theta }{\omega },$$

where *ω*_*v*_ is the vertex angular frequency corresponding to *k*_*v*_.

**Figure 4:**
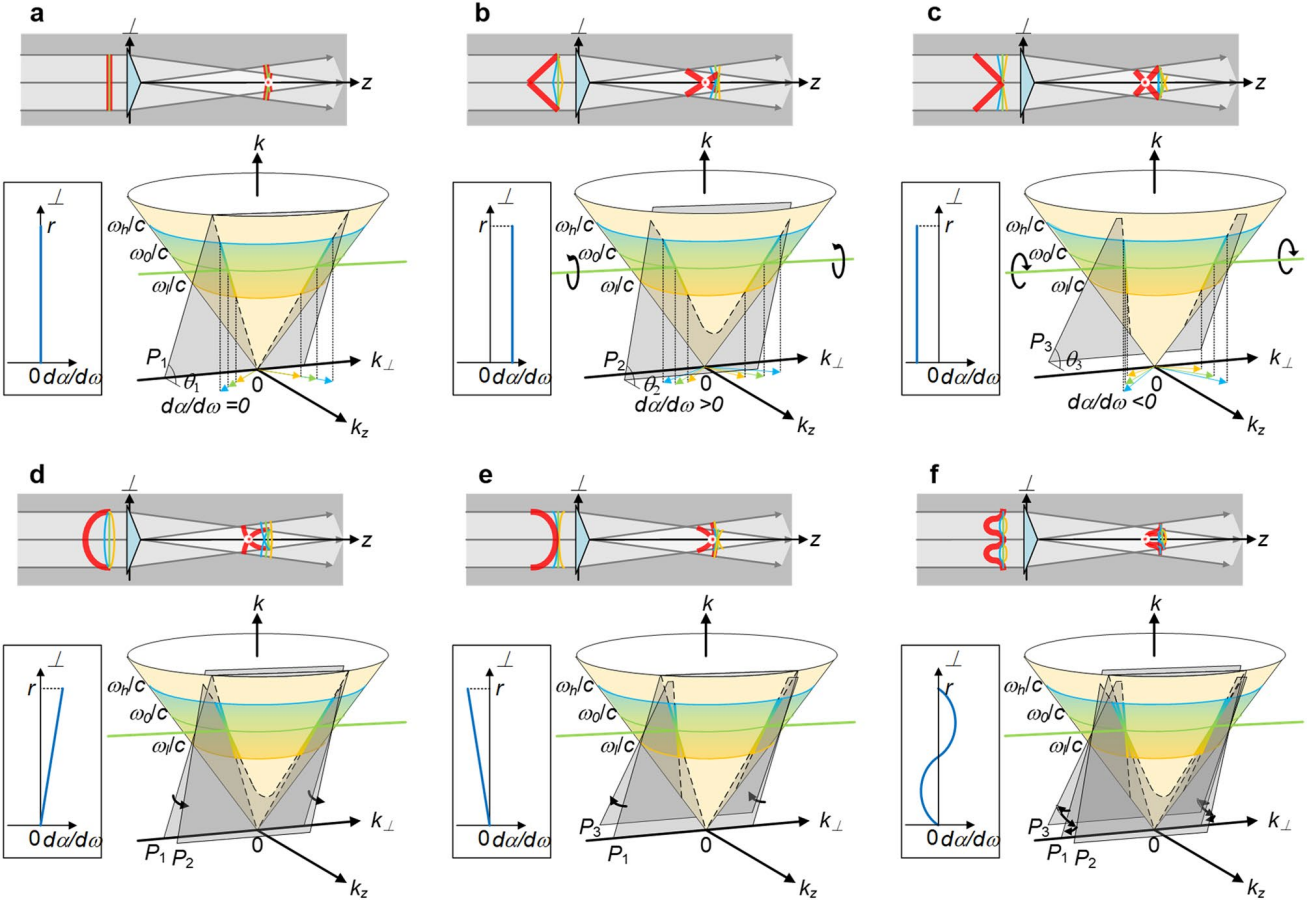
Group-velocity-tunable propagation-invariant optical wave-packet generated by the conical superposition and its spatiotemporal spectrum in the Fourier space. Spatiotemporal spectrum (colored thick line) of propagation-invariant optical wave-packet is a part of intersecting curve between light-cone and spectral plane, and spectral plane is parallel to *k*_*⊥*_-axis and passes through green thick straight line (*k*_*z*_, *k*) = (*ω*_*0*_/*c*⋅cos*α*_0_, *ω*_*0*_/*c*). Vertex is defined as intersecting point between spatiotemporal spectrum’s locus and plane *k*_*⊥*_ = 0 at positive direction of *k*_*z*_-axis. (**a**) Without pulse-front deformation, no angular dispersion across beam aperture *dα*/*dω* = 0, vertex is at origin, and spectral plane is *P*_1_ with tilt angle *θ*_1_; (**b**) with concave-conical pulse-front deformation, constant positive angular dispersion across beam aperture *dα*/*dω* > 0, vertex is *k*_*v*_ < *ω*_*l*_/*c*, and spectral plane is *P*_2_ with tilt angle *θ*_2_; (**c**) with convex-conical pulse-front deformation, constant negative angular dispersion across beam aperture *dα*/*dω* < 0, vertex is *k*_*v*_ > *ω*_*h*_/*c*, and spectral plane is *P*_3_ with tilt angle *θ*_3_; (**d**) with concave-spherical pulse-front deformation, from beam center to upper beam edge, angular dispersion *dα*/*dω* increases linearly from zero to maximum, and instantaneous spectral plane is rotating counterclockwise from *P*_1_ to *P*_2_; (**e**) with convex-spherical pulse-front deformation, from beam center to upper beam edge, angular dispersion *dα*/*dω* decreases linearly from zero to minimum, and instantaneous spectral plane is rotating clockwise from *P*_1_ to *P*_3_; and (**f**) with cosine-function-like pulse-front deformation, from beam center to upper beam edge, angular dispersion *dα*/*dω* decreases from zero to minimum, increases to zero and then maximum, and decreases to zero eventually, and instantaneous spectral plane is rotating clockwise from *P*_1_ to *P*_3_, counterclockwise to *P*_1_ and then *P*_2_, and clockwise to *P*_1_ eventually. Tilt angles are *θ*_3_ < *θ*_1_ < *θ*_2_; *k*_*v*_ is wavenumber of vertex; *ω*_*l*_, *ω*_*0*_, and *ω*_*h*_ are the lowest, center and highest angular frequencies of pulse; and *r* is beam radius. Figure generated by Microsoft® Visio® 2013^50^.

Because the collimation by the retroreflection mirror keeps the phase-front (wave-front of the center angular frequency *ω*_*0*_) to the thin axicon always flat, a given thin axicon fixes the half conical-angle of the center angular frequency *ω*_*0*_ to a constant *α*_0_, that is the spectral plane of the optical wave-packet must pass through a fixed straight line (*k*_*z*_, *k*) = (*ω*_*0*_/*c*⋅cos*α*_0_, *ω*_*0*_/*c*), as illustrated by the green thick straight line in Fig. 4. Then, once the vertex is determined, the tilt angle *θ* of the spectral plane, as well as the spatiotemporal spectrum, would be determined. When the vertex is at the origin (*k*_*⊥*_, *k*_*z*_, *k*) = (0, 0, 0), Fig. 4a shows the spectral plane is *P*_1_ with a tilt angle *θ*_*1*_, and Eq. (12) is simplified to tan*θ*_*1*_ = 1/cos*α*. The tilt angle *θ*_*1*_ of the spectral plane *P*_1_ in the Fourier space is directly linked with the half conical-angle *α* in the physical space, and *α* = *α*_*0*_ is a constant. Figure 4a also shows there is no angular dispersion *dα*/*dω* = 0 both in the *⊥*-z plane in the physical space and in the *k*_*⊥*_-*k*_*z*_ plane in the Fourier space, and we use a *dα*/*dω*-*⊥* plot to describe the angular dispersion variation across the input beam aperture (from the beam center to the upper beam edge). The spatiotemporal spectrum consists of two parts and lies in two straight lines through the two fixed points (*k*_*x*_, *k*_*z*_, *k*) = (± *ω*_*0*_/*c*⋅sin*α*_0_, *ω*_*0*_/*c*⋅cos*α*_0_, *ω*_*0*_/*c*) and the vertex (*k*_*x*_, *k*_*z*_, *k*) = (0, 0, 0). *ω*_*l*_, *ω*_*0*_, *ω*_*h*_ are the lowest, center and highest angular frequencies of the pulse and are illustrated by yellow, green and blue circles on the light cone. If the vertex of the spatiotemporal spectrum is not at the origin, Fig. 4b shows when the spectral plane is rotated counterclockwise about the fixed straight line (*k*_*z*_, *k*) = (*ω*_*0*_/*c*⋅cos*α*_0_, *ω*_*0*_/*c*) from *P*_1_ to *P*_2_ with a tilt angle *θ*_*2*_, the tilt angle of the spectral plane and accordingly the group-velocity of the propagation-invariant optical wave-packet are increased, and a constant positive conical-angle dispersion *dα*/*dω* > 0 appears across the beam aperture which is also illustrated by wavefronts in the *⊥*-z plane in the physical space and wavevectors in the *k*_*⊥*_-*k*_*z*_ plane in the Fourier space. Similarly, Fig. 4c shows when the spectral plane is rotated clockwise about the fixed straight line (*k*_*z*_, *k*) = (*ω*_*0*_/*c*⋅cos*α*_0_, *ω*_*0*_/*c*) from *P*_1_ to *P*_3_ with a tilt angle *θ*_*3*_, the tilt angle of the spectral plane and accordingly the group-velocity of the propagation-invariant optical wave-packet are reduced, and a constant negative conical-angle dispersion *dα*/*dω* < 0 appears across the beam aperture which is also illustrated by wavefronts in the *⊥*-z plane in the physical space and wavevectors in the *k*_*⊥*_-*k*_*z*_ plane in the Fourier space.

By differentiating two sides of Eq. (12) about the center angular frequency *ω*_0_, the conical-angle dispersion is given by

13 $$\frac{d\alpha }{d\omega }=\frac{\left(1-cot\theta \right)}{sin{\alpha }_{0}}\frac{{\omega }_{v}}{{\omega }_{0}^{2}}.$$

From Eq. (12), the vertex angular frequency *ω*_*v*_ can be described by

14 $${\omega }_{v}=\frac{{\omega }_{0}\left(cos{\alpha }_{0}-cot\theta \right)}{1-cot\theta }.$$

By the substitution of Eq. (13) with Eq. (14), we have

15 $$tan\theta =\frac{1}{cos{\alpha }_{0}-{\omega }_{0}\frac{d\alpha }{d\omega }sin{\alpha }_{0}}.$$

Equation (15) shows the tilt angle *θ* of the spectral plane can be changed by introducing and then adjusting a non-zero conical-angle dispersion *dα*/*dω*. Equations (15) and (9) link the conical-angle dispersion *dα*/*dω* with the tilt angle *θ* of the spectral plane and accordingly with the group-velocity *v*_g_ of the propagation-invariant optical wave-packet. Because the half conical-angle *α*_0_ usually is very small, the tilt angle *θ*, and accordingly the group-velocity *v*_g_, increases with increasing the conical-angle dispersion *dα*/*dω*, and vice versa.

When the conical-angle dispersion is not a constant and becomes space-dependent across the beam aperture *dα*(*⊥*)/*dω* (but still axisymmetric about the *z*-axis), the tilt angle *θ*(*z*) of an instantaneous spectral plane in the Fourier space, which corresponds to an instantaneous propagation-invariant optical wave-packet propagating at the *z*-axis in the physical space, should be revised as

16 $$tan\theta \left(z\right)=\frac{1}{cos{\alpha }_{0}-{\omega }_{0}\frac{d\alpha \left(\perp \right)}{d\omega }sin{\alpha }_{0}},$$

where, *dα*(*⊥*)/*dω* is the variable conical-angle dispersion along the *⊥*-axis at the input, and the transverse and longitudinal coordinates satisfy the conical superposition relationship *⊥*/*z* = tan*α*_*0*_. Figure 4d illustrates when the input has a concave-spherical pulse-front deformation, from the beam center to the upper beam edge the conical-angle dispersion *dα*(*⊥*)/*dω* increases linearly from zero to the maximum, and during propagation of the propagation-invariant optical wave-packet, the corresponding tilt angle *θ*(*z*) of the instantaneous spectral plane in the Fourier space is rotating counterclockwise from *P*_1_ to *P*_2_, showing accelerating group-velocity. Figure 4e illustrates when the input has a convex-spherical pulse-front deformation, from the beam center to the upper beam edge the conical-angle dispersion *dα*(*⊥*)/*dω* decreases linearly from zero to the minimum, and during propagation of the propagation-invariant optical wave-packet, the corresponding tilt angle *θ*(*z*) of the instantaneous spectral plane in the Fourier space is rotating clockwise from *P*_1_ to *P*_3_, showing decelerating group-velocity. Essentially, the acceleration/deceleration of the propagation-invariant optical wave-packet here and that of the space–time wave-packet reported by M. Yessenov and L. Hall et al.^36,55^ are the same. Each temporal frequency *ω* is associated not with a single spatial frequency *k*_*⊥*_(*ω*) but a variable one [*k*_*⊥*_(*ω*, *z* = 0), *k*_*⊥*_(*ω*, *z* = *L*)], where *L* is the propagation distance of the wave-packet. Figure 4f illustrates when the input has an axisymmetric cosine-function-like pulse-front deformation, from the beam center to the upper beam edge the conical-angle dispersion *dα*(*⊥*)/*dω* decreases from zero to the minimum, increases to zero and then maximum, and finally decreases to zero again, and during propagation of the propagation-invariant optical wave-packet, the corresponding tilt angle *θ*(*z*) of the instantaneous spectral plane in the Fourier space is rotating clockwise from *P*_1_ to *P*_3_, counterclockwise to *P*_1_ and then *P*_2_, and finally clockwise to *P*_1_ again, showing periodically variable (decelerating-accelerating-decelerating) group-velocity. If neglecting the diffraction distortion, the variation of the tilt angle *θ*(*z*) of the instantaneous spectral plane in the Fourier space, which corresponds to a propagating propagation-invariant optical wave-packet, can be described by Eq. (16). However, if the conical-angle dispersion *dα*(*⊥*)/*dω* is too large, the geometrical approximation is destroyed by the propagation diffraction and the angular-dispersion-induced distortion, and the propagation-invariant optical wave-packet would distort during propagation^47^. Then, the tunable ranges of the group-velocity and the group-acceleration are limited, typically only around 0.01*c* (m/s) and ± 0.3*c*^2^ (m/s^2^), respectively, and the propagation distance is also limited, usually around 100 mm^46^.

## Extension and discussion

In the physical space, the tunable group-velocity *v*_g_ of the propagation-invariant optical wave-packet generated by the conical superposition is due to the pulse-front tilt angle *β*, which is caused by the conical-angle dispersion *dα*/*dω*. In the Fourier space, the tunable group-velocity *v*_g_ of this propagation-invariant optical wave-packet is due to the tilt angle *θ* of the spectral plane, which is also caused by the conical-angle dispersion *dα*/*dω*. If substituting Eq. (15) with Eq. (6), Eq. (15) becomes Eq. (7), which shows the group-velocity equation derived in the Fourier space and that derived in the physical space are exactly equivalent. Then, the nature of controlling the group-velocity of this propagation-invariant optical wave-packet is introducing and then adjusting the conical-angle dispersion across the input beam aperture. It is worth mentioning that the conical-angle dispersion should be axisymmetric about the z-axis for straight-line propagation.

**Figure 5:**
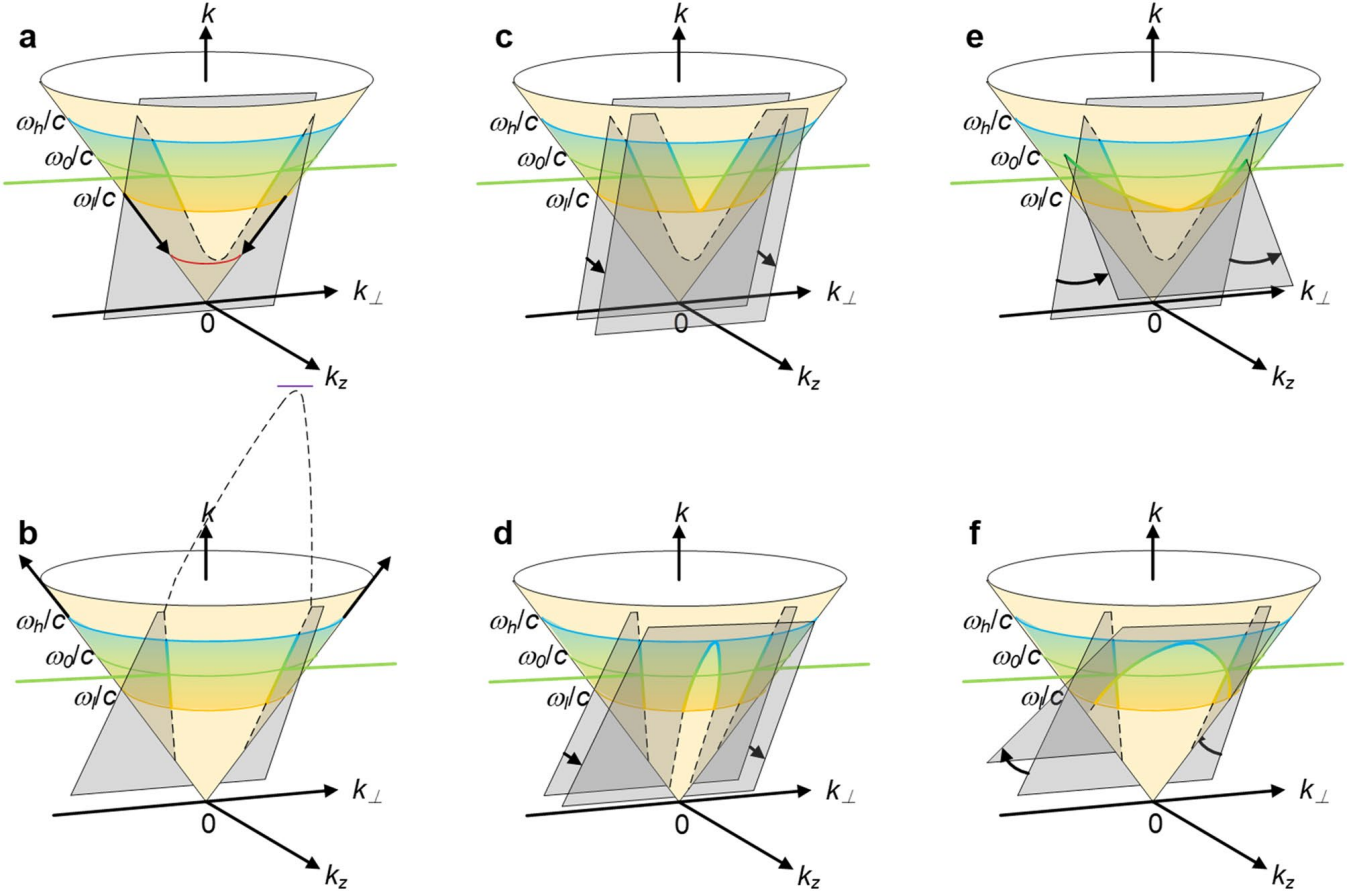
Group-velocity-tunable propagation-invariant optical wave-packet generated by the conical superposition is subset of space–time wave-packet. Spatiotemporal spectrum (colored thick line on light-cone) of propagation-invariant optical wave-packet generated by the conical superposition is a part of that of space–time wave-packet, excluding *k*_*⊥*_ = 0. When (**a,b**) spectral range of pulse is enhanced, or spectral plane is (**c,d**) shifted towards positive direction of *k*_z_-axis, or spectral plane is dramatically rotated (**e**) counterclockwise or (**f**) clockwise, spatiotemporal spectrum of propagation-invariant optical wave-packet is changed from two separate curves about *k*_*⊥*_ = 0 plane into a single continuous one, and propagation-invariant optical wave-packet becomes space–time wave-packet. *ω*_*l*_, *ω*_*0*_, and *ω*_*h*_ are the lowest, center and highest angular frequencies of pulse. Figure generated by Microsoft® Visio® 2013^50^.

Figure 5 shows, in the Fourier space, the spatiotemporal spectrum of this propagation-invariant optical wave-packet consists of two separate lines (excluding *k*_*⊥*_ = 0) lying in the intersecting curve between the light-cone and the spectral plane parallel to the *k*_*⊥*_-axis, which are symmetric about the *k*-*k*_z_ plane. The spatiotemporal spectrum of the space–time wave-packet, well studied by Abouraddy et al.^30–37^, is a single continuous conic curve (including *k*_*⊥*_ = 0) lying in the intersecting curve between the light-cone and the spectral plane parallel to the *k*_*⊥*_-axis. Consequently, in the Fourier space, this group-velocity-tunable propagation-invariant optical wave-packet is a part of the space–time wave-packet, and then the former should be a subset of the latter.

Figure 5a,b show if we can significantly increase the spectral range of the pulse, especially the lowest angular frequency *ω*_*l*_ and the highest angular frequency *ω*_*h*_ for the case of the increased (superluminal) and the reduced (subluminal) group-velocity, respectively, the spatiotemporal spectrum can be changed from two separate lines into a single continuous conic curve, and the propagation-invariant optical wave-packet generated by the conical superposition would be improved to the space–time wave-packet. Besides, Fig. 5c,d show, no matter for the case of the group-velocity increased (superluminal) or reduced (subluminal) propagation-invariant optical wave-packet, we can shift the spectral plane towards the positive direction of the *k*_z_-axis to make the spatiotemporal spectrum continuous within the spectral range of the pulse, for example by dramatically reducing the conical angle generated by the thin axicon to near zero. In addition, Fig. 5e,f show we can also rotate the spectral plane about the fixed straight line (*k*_*z*_, *k*) = (*ω*_*0*_/*c*⋅cos*α*_0_, *ω*_*0*_/*c*) counterclockwise and clockwise for the case of the group-velocity increased (superluminal) and reduced (subluminal) propagation-invariant optical wave-packet, respectively, to produce a continuous spatiotemporal spectrum within the spectral range of the pulse, for example by increasing the absolute value of the conical-angle dispersion. In the above processes of changing the spatiotemporal spectrum from two separate lines into a single continuous conic curve, the classic optics we used in Fig. 1 are not enough, and elements like SLM, just as A. Abouraddy et al. have done, are necessary.

## Conclusion

In conclusion, we have systematically introduced different forms of the group-velocity-tunable propagation-invariant optical wave-packet generated by the conical superposition. The classic optical setups for generating propagation-invariant optical wave-packets with superluminal, subluminal, accelerating, decelerating, and near-programmable group-velocities are also introduced. The mechanism of the tunability of the group-velocity is analyzed in the physical space and the Fourier space, respectively, and the group-velocity equations are derived in two spaces and also linked in mathematics. In the physical space, this propagation-invariant optical wave-packet is generated by the conical superposition of pulse-fronts, which at different propagating locations have different instantaneous pulse-front tilt angles and accordingly have different instantaneous group-velocities. While, in the Fourier space, the spatiotemporal spectrum of this propagation-invariant optical wave-packet lies in the intersecting curve between the light-cone and a spectral plane parallel to the *k*_*⊥*_-axis, and the tilt angle of the spectral plane determines the group-velocity. The propagation-invariant optical wave-packets at different propagating locations correspond to different spectral planes with different tilt angles and consequently different instantaneous group-velocities. However, both the pulse-front tilt angle in the physical space and the spectral plane tilt angle in the Fourier space are due to the angular dispersion, which actually is the nature of controlling the group-velocity of this propagation-invariant optical wave-packet.

In the Fourier space, because the spatiotemporal spectrum of this group-velocity-tunable propagation-invariant optical wave-packet is a part of that of the space–time wave-packet, the former should be a subset of the latter. Due to the generation methods only using classic optics, the spatiotemporal spectrum of this group-velocity-tunable propagation-invariant optical wave-packet consists of two separate and symmetric short lines. When increasing the spectral range of the pulse, or shifting the spectral plane in the Fourier space, or rotating the spectral plane in the Fourier space, the spatiotemporal spectrum could be changed from two separate lines into a single continuous conic curve, and accordingly this group-velocity-tunable propagation-invariant optical wave-packet would be improved to the space–time wave-packet.

## Data Availability

All data and models generated or used during the study appear in the submitted article.
